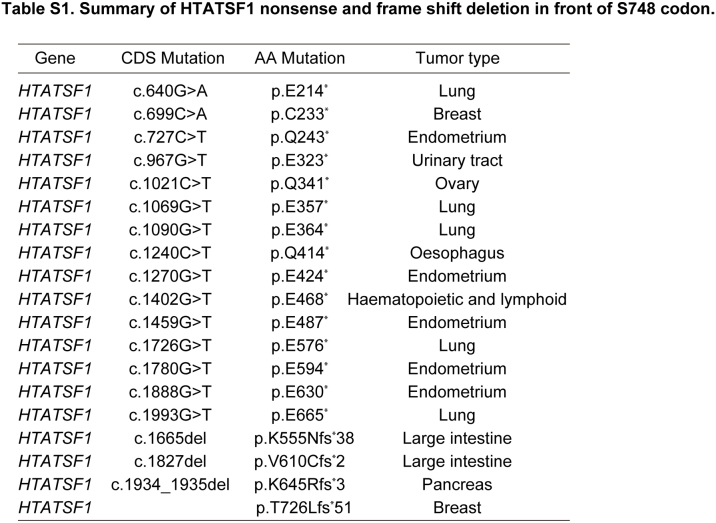# CK2–HTATSF1–TOPBP1 signaling axis modulates tumor chemotherapy response

**DOI:** 10.1016/j.jbc.2024.107377

**Published:** 2024-05-16

**Authors:** Qiushi Guo, Jiao Zhao, Yuan Li, Chunyong Zhang, Xilin Shen, Ling Liu, Zhenzhen Yang, Shuai Ma, Yan Qin, Lei Shi

**Affiliations:** 1 Key Laboratory of Breast Cancer Prevention and Therapy (Ministry of Education), Key Laboratory of Immune Microenvironment and Disease (Ministry of Education), The Province and Ministry Co-sponsored Collaborative Innovation Center for Medical Epigenetics, School of Basic Medical Sciences, Tianjin Medical University Cancer Institute and Hospital, Tianjin Medical University, Tianjin, China; 2 Key Clinical Laboratory of Henan Province, Department of Clinical Laboratory, Zhengzhou University, The First Affiliated Hospital of Zhengzhou University, Zhengzhou, China

**Keywords:** breast cancer, genomic stability, DNA damage, homologous recombination, CK2 HTATSF1–TOPBP1

## Abstract

Homologous recombination (HR) plays a key role in maintaining genomic stability, and the efficiency of the HR system is closely associated with tumor response to chemotherapy. Our previous work reported that CK2 kinase phosphorylates HIV Tat-specific factor 1 (HTATSF1) Ser748 to facilitate HTATSF1 interaction with TOPBP1, which in turn, promotes RAD51 recruitment and HR repair. However, the clinical implication of the CK2–HTATSF1–TOPBP1 pathway in tumorigenesis and chemotherapeutic response remains to be elucidated. Here, we report that the CK2–HTATSF1–TOPBP1 axis is generally hyperactivated in multiple malignancies and renders breast tumors less responsive to chemotherapy. In contrast, deletion mutations of each gene in this axis, which also occur in breast and lung tumor samples, predict higher HR deficiency scores, and tumor cells bearing a loss-of-function mutation of *HTATSF1* are vulnerable to poly(ADP-ribose) polymerase inhibitors or platinum drugs. Taken together, our study suggests that the integrity of the CK2–HTATSF1–TOPBP1 axis is closely linked to tumorigenesis and serves as an indicator of tumor HR status and modulates chemotherapy response.

Homologous recombination (HR) deficiency (HRD) due to germline or somatic mutation of HR genes is a common feature of many tumors (1, 2). There is a large body of literature suggesting that HRD is associated with a specific phenotype characterized by sensitivity to poly(ADP-ribose) polymerase inhibitors (PARPis) or platinum-based therapies, which confers synthetic lethality to HRD cancers and undoubtedly improves prognosis (2, 3, 4, 5). However, breast and ovarian cancers carrying germline *BRCA1* and *BRCA2* gene mutations, which have been deemed archetypal in determining an HRD phenotype, can gradually acquire HR activity following PARPi therapy (6). In addition, a large number of patients with HR proficiency tumors may not benefit from these drugs (6, 7, 8, 9). Therefore, despite promising clinical results, overcoming *de novo* and acquired HR activity remains one of the major challenges for PARPi therapy. These events call for an understanding of the biological impact of individual aberrations in many of the HR genes, including mutations, deletions, and amplifications, which will help to predict the outcome of PARPi or platinum-based chemotherapy and expand the potential use of these drugs.

HR requires the spatiotemporal orchestration of repair machinery in specific genomic environments and biological contexts. Briefly, it begins with a 5′-3′ end resection to generate a 3′-overhanged single-stranded DNA. The single-stranded DNA is then bound by replication protein A (RPA), which is subsequently replaced by RAD51 to form a nucleofilament that invades the sister chromatid or homologous template to copy genetic information (10). TOPBP1, a scaffold protein containing multiple BRCA1 C-terminal domains, has been reported to promote PLK1-mediated phosphorylation of RAD51, thereby facilitating RAD51 filament formation (11). Recently, we reported that CK2 kinase-mediated Ser748 phosphorylation (pS748) of HIV Tat-specific factor 1 (HTATSF1) facilitates HTATSF1 binding to TOPBP1 at RPA-coated postresected double-strand break ends, where the HTATSF1–TOPBP1 complex further promotes RAD51 loading and RPA clearance (12).

TOPBP1 is overexpressed in multiple malignancies, including breast cancer, lung cancer, and ovarian cancer (13). Accordingly, increased levels of TOPBP1 expression make cells more resistant to radiotherapy, increase the risk of relapse or death, and lead to poor survival (11, 14, 15, 16, 17, 18). Disruption of TOPBP1 in these cancer cells results in increased sensitivity to radiation or PARPi (11, 15, 16), and mutation of TOPBP1 in clinical tumor samples impairs its ability to recruit to and repair of DNA lesions (19). Dysregulated expression of HTATSF1 has also been reported in lung cancer compared to normal adjacent tissues (20). Thereby, it is of interest to understand the significance of HTATSF1–TOPBP1 complex–mediated HR repair during cancer progression and for therapeutic implications.

In this study, we report that the CK2–HTATSF1–TOPBP1 axis is overactivated in a number of tumors, with concomitant amplification and upregulation of *CSNK2A1*, *HTATSF1*, and *TOPBP1*. pS748, a CK2-driven HTATSF1 phosphorylation site, is also significantly elevated and correlates with breast tumor progression. Notably, disruption of HTATSF1–TOPBP1 binding in CK2–HTATSF1–TOPBP1 proficient tumors leads to DNA damage accumulation, breast tumor suppression, and increased sensitivity to chemotherapy. In addition, HTATSF1 truncations or mutations, although less common than amplification, increase the sensitivity of breast and lung tumor cells to PARPi and platinum drug, suggesting potential synthetic lethality strategies for HTATSF1-deficient or -mutant cancers. Our findings support targeting CK2–HTATSF1–TOPBP1 as a potential therapeutic avenue for proficient tumors and highlight loss-of-function of the pathway as a therapeutic indicator for PARPi and platinum drugs.

## Results

### The CK2–HTATSF1–TOPBP1 signaling axis is generally overactivated among multiple tumors

**Figure 1:**
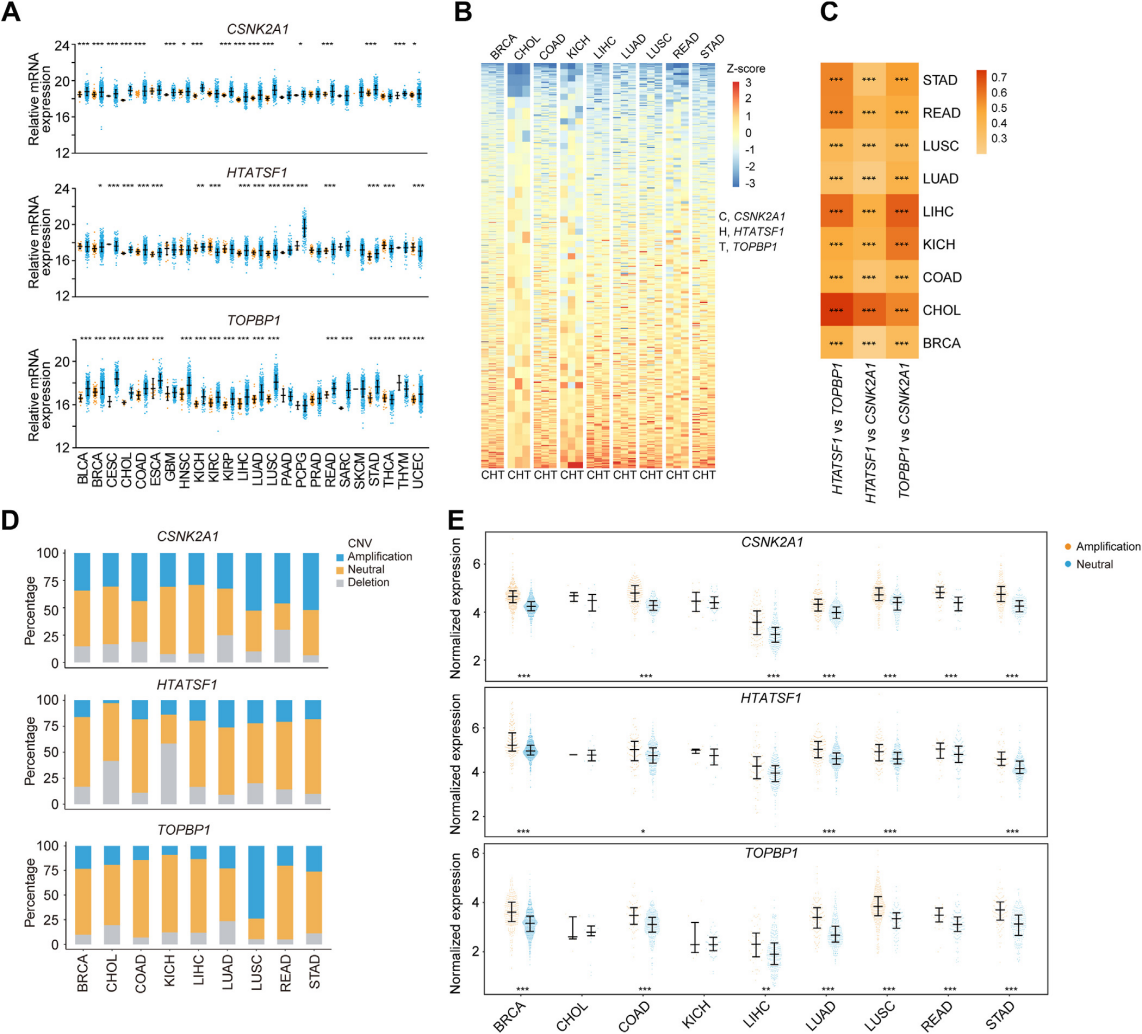
**Human *CSNK2A1*, *HTATSF1*, and *TOPBP1* are upregulated in multiple tumors.***A*, analysis of the expression of *CSNK2A1*, *HTATSF1*, and *TOPBP1* in 24 different tumor types and the corresponding tumor-adjacent normal tissues from the TCGA datasets. *B*, the mRNA expression profile of *CSNK2A1*, *HTATSF1*, and *TOPBP1* in nine tumor types as indicated by TCGA datasets. The color key to the Z-score represents the normalized expression. *C*, the mRNA expression correlation between each two of *CSNK2A1*, *HTATSF1*, and *TOPBP1* in nine types of TCGA tumors. The color key represents Pearson correlation coefficient. *D*, copy number variation analysis of *CSNK2A1*, *HTATSF1*, and *TOPBP1* in nine TCGA tumor types where the three genes are highly expressed at mRNA level in these tumors. *E*, analysis of the correlation between the mRNA expression and amplification of *CSNK2A1*, *HTATSF1*, and *TOPBP1* in nine types of TCGA tumor. Data are mean ± SD for (*A*) and (*E*). ∗*p* < 0.05, ∗∗*p* < 0.01, ∗∗∗*p* < 0.001; Mann-Whitney test for (*A*) and (*E*). Two-tailed unpaired Student’s *t* test for (*C*). BLCA, bladder urothelial carcinoma; BRCA, breast invasive carcinoma; CESC, cervical squamous cell carcinoma and endocervical adenocarcinoma; CHOL, cholangiocarcinoma; COAD, colon adenocarcinoma; ESCA, esophageal carcinoma; GBM, glioblastoma multiforme; HNSC, head and neck squamous cell carcinoma; HTATSF1, HIV Tat-specific factor 1; KICH, kidney chromophobe; KIRC, kidney renal clear cell carcinoma; KIRP, kidney renal papillary cell carcinoma; LIHC, liver hepatocellular carcinoma; LUAD, lung adenocarcinoma; LUSC, lung squamous cell carcinoma; PAAD, pancreatic adenocarcinoma; PCGP, pheochromocytoma and paraganglioma; PRAD, prostate adenocarcinoma; READ, rectum adenocarcinoma; SARC, sarcoma; SKCM, skin cutaneous melanoma; STAD, stomach adenocarcinoma; THCA, thyroid carcinoma; THYM, thymoma; UCEC, uterine corpus endometrial carcinoma; NS, not significant; TCGA, The Cancer Genome Atlas.

Tumors, characterized by rapid, unregulated cell division and genome instability, rely heavily on HR to repair damaged DNA resulting from replication stress and are inherently vulnerable to exogenous DNA damage or DDR inhibitors (21, 22). Therefore, we wondered whether the CK2–HTATSF1–TOPBP1 axis is dysregulated in tumors and involved in tumorigenesis. To test this hypothesis, we first evaluated the expression of *CSNK2A1*, which encodes the catalytic subunit of CK2, *HTATSF1*, and *TOPBP1*, using The Cancer Genome Atlas (TCGA) datasets. The results showed that the mRNA expression levels of *CSNK2A1*, *HTATSF1*, and *TOPBP1* are simultaneously upregulated in 9 out of 24 types of malignancies (Fig. 1*A*), and the expression of these genes is positively correlated with each other (Fig. 1, *B* and *C*). These results raise the possibility that the CK2–HTATSF1–TOPBP1 signaling pathway may be overactivated in multiple tumors.

We also showed that the mRNA level of *CSNK2A1*, *HTATSF1*, and *TOPBP1* is largely positively correlated with that of *PCNA* and *MKI67* among multiple tumors from the TCGA datasets (Fig. S1, *A* and *B*), suggesting that the CK2–HTATSF1–TOPBP1 axis is associated with higher proliferating signatures. Next, copy number variation analysis revealed that each gene in this pathway gains copy number, albeit to a different extent (Fig. 1*D*). The amplification of each gene largely correlates with its expression in tumors from breast, colon, lung, and gastric tissues (Fig. 1*E*), suggesting that the upregulation of CSNK2A1, HTATSF1, and TOPBP1 is likely to be a result of gene amplification in these tumors.

### The HTATSF1 pS748 is upregulated in breast tumor

**Figure 2:**
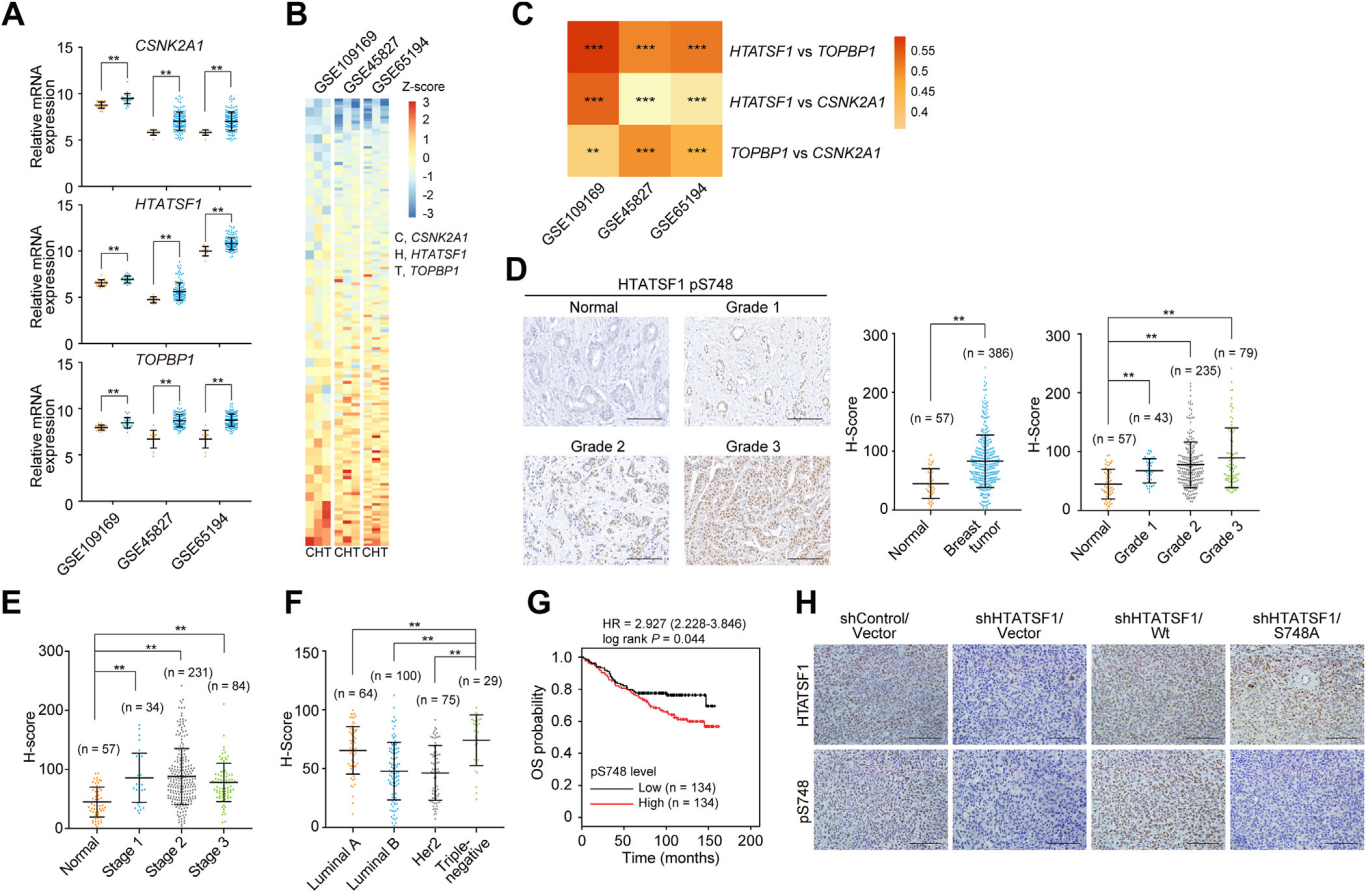
**HTATSF1 pS748 is upregulated in breast tumor.***A*, analysis of the expression of *CSNK2A1*, *HTATSF1*, and *TOPBP1* in breast cancer tissues and the corresponding tumor-adjacent normal tissues from three independent GEO datasets. *B*, the expression profile of *CSNK2A1*, *HTATSF1*, and *TOPBP1* in breast tumor from three independent GEO datasets. The color key to the Z-score represents the normalized expression. *C*, the expression correlation between each two of *CSNK2A1*, *HTATSF1*, and *TOPBP1* in breast tumor from three independent datasets of GEO. The color key represents Pearson correlation coefficient. *D*, immunohistochemistry (IHC) analysis of HTATSF1 pS748 in tumor-adjacent normal breast tissue and different grades of breast cancer samples. Representative images are shown. The level of HTATSF1 pS748 from IHC staining was scored according to the staining intensity. *E*, quantitative analysis of HTATSF1 pS748 IHC signals in tumor-adjacent normal breast tissue and different stages of breast cancer samples. *F*, quantitative analysis of HTATSF1 pS748 levels in different molecular subtypes of breast cancer. *G*, overall survival (OS) analysis of breast cancer patients according to the level of HTATSF1 pS748. *H*, IHC analysis of xenograft tumor tissue from MDA-MB-231 cells expressing the indicated shRNAs (shHTATSF1 targeting the 3′UTR of *HTATSF1*) and different HTATSF1 variants using antibodies against HTATSF1 and HTATSF1 pS748. Scale bar, 100 μm. Data are mean ± SD for (*D–F*) from biological triplicate experiments. ∗∗*p* < 0.01, ∗∗∗*p* < 0.001; NS, not significant; Mann-Whitney test for (*A*), (*D*), (*E*) and (*F*); Two-tailed unpaired Student’s *t* test for (*C*); Log-rank test for (*G*). GEO, Gene Expression Omnibus; HTATSF1, HIV Tat-specific factor 1.

As both CK2 and TOPBP1 have been implicated in breast tumorigenesis (15, 23, 24), we then aimed to evaluate the role of CK2–HTATSF1–TOPBP1 axis in breast tumor. First, we confirmed the expression and correlation of *CSNK2A1*, *HTATSF1*, and *TOPBP1* with three independent datasets from Gene Expression Omnibus (GEO) (Fig. 2, *A–C*). We then analyzed the level of HTATSF1 pS748, which is a functional readout of CK2 activity and is interpreted by TOPBP1, *via* immunohistochemistry (IHC) in breast tumor tissue and tumor-adjacent normal breast tissue. Consistent with the expression profile of *CSNK2A1* and *HTATSF1* from the TCGA and GEO data, quantitative analysis of IHC staining showed that the level of pS748 was significantly elevated in carcinoma samples, and it is positively correlated with the malignant degree (grades) of breast cancer samples (Fig. 2*D*), although it does not well reflect tumor stages, a comprehensive index of primary tumor size and distant metastasis (Fig. 2*E*). Further analysis revealed that triple-negative breast cancer showed the strongest staining of pS748 among different molecular subtypes (Fig. 2*F*), and higher levels of pS748 predicted worse outcome in breast cancer patients (Fig. 2*G*). The specificity of pS748 antibody was confirmed using xenograft tumor tissue from MDA-MB-231 cells expressing HTATSF1 shRNA and wildtype HTATSF1 (HTATSF1/Wt) or alanine-substituted mutant HTATSF1/S748A (Fig. 2*H*). These data suggest a potentially important role for pS748 in breast tumorigenesis.

In agreement with our previous report that HTATSF1 is enriched S phase of the cell cycle, immunostaining followed by confocal microscopy analysis showed that HTATSF1 is highly expressed in EdU-labeled MDA-MB-231 cells and BT-549 cells (Fig. S2, *A* and *B*). Similar observations were obtained when examining the level of HTATSF1 pS748 in these breast cancer cells (Fig. S2, *A* and *B*). It is likely that in the S-phase of cell cycle, highly expressed HTATSF1 allows actively replicating breast cancer cells to be well-equipped with HTATSF1 pS748-orchestrated CK2–HTATSF1–TOPBP1 axis, thus counteracting replication stress-induced DSBs *via* HR repair.

### CK2–HTATSF1–TOPBP1 axis protects breast cancer cells from genotoxic insults

**Figure 3:**
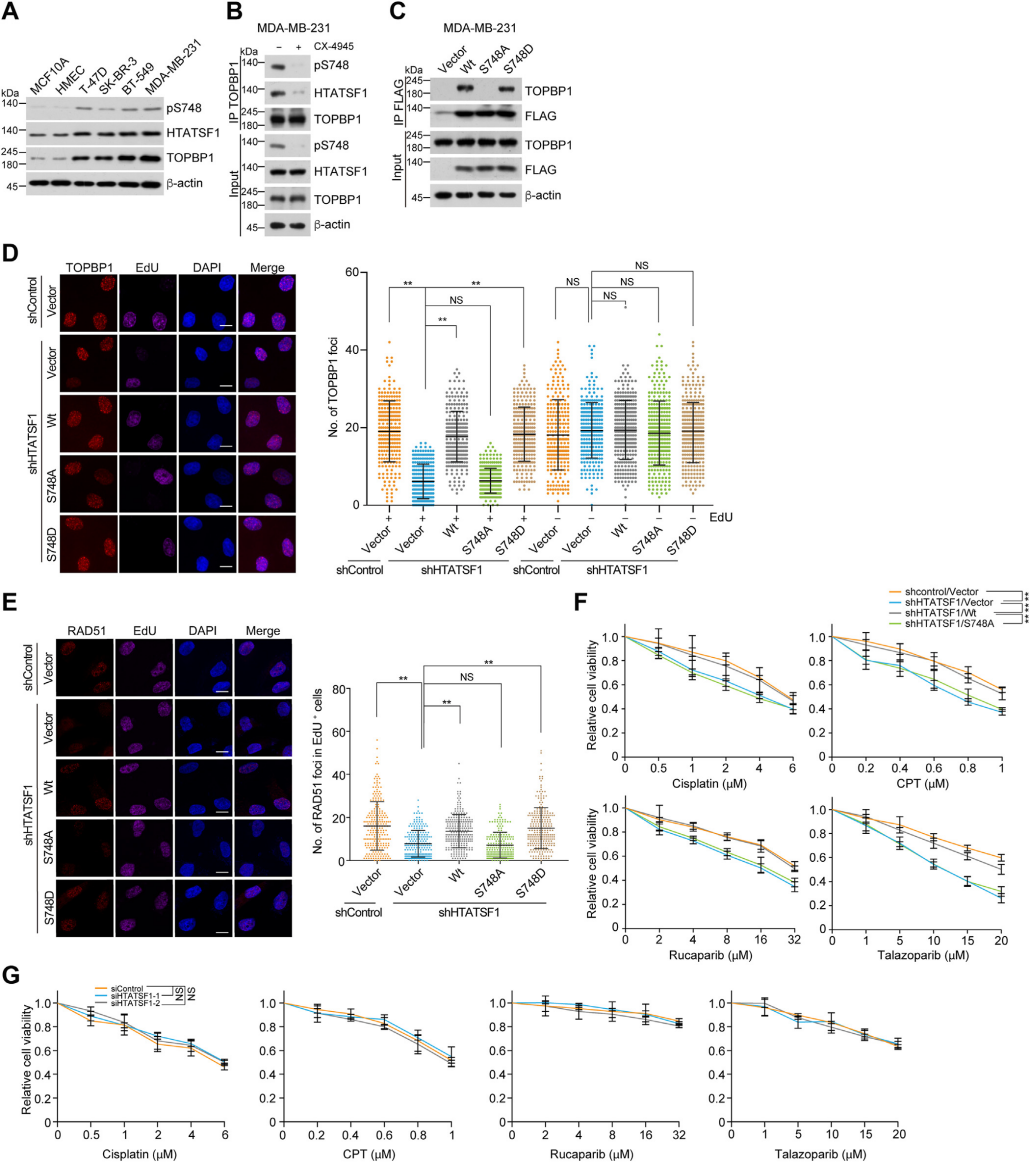
**CK2–HTATSF1–TOPBP1 axis protects breast cancer cells from genotoxic insults.***A*, immunoblotting analysis of HTATSF1 pS748 level, HTATSF1, and TOPBP1 expression in MCF10A, HMEC, and breast cancer cells with different molecular subtypes. *B*, immunoprecipitation and immunoblotting with cellular extracts from MDA-MB-231 cells treated with CK2 inhibitor CX-4945 (10 μM, 4 h). *C*, co-immunoprecipitation analysis of the interaction between endogenous TOPBP1 and the indicated point mutants of FLAG-tagged HTATSF1. *D*, immunostaining and confocal microscopy analysis of TOPBP1 foci formation in MDA-MB-231 cells. Cells stably expressing HTATSF1 3′UTR shRNA and HTATSF1/Wt, HTATSF1/S748A, or HTATSF1/S748D, were treated with cisplatin (20 μM, 8 h) and labeled with EdU for 1 h followed by pre-extraction and fixation. The foci number in EdU-positive and EdU-negative cells was quantified (n > 230). Scale bar, 10 μm. *E*, immunostaining and confocal microscopy analysis of RAD51 foci formation in MDA-MB-231 cells. Cells stably expressing HTATSF1 3′UTR shRNA and HTATSF1/Wt, HTATSF1/S748A, or HTATSF1/S748D were treated with cisplatin (20 μM, 8 h) and labeled with EdU for 1 h before collection. The foci number in EdU-positive cells was quantified (n > 240). Scale bar, 10 μm. *F*, survival analysis of MDA-MB-231 cells expressing HTATSF1 3′ UTR shRNA and HTATSF1/Wt or HTATSF1/S748A under different drug treatments. *G*, survival analysis of HMECs expressing HTATSF1 siRNAs under different drug treatments. Data are mean ± SD for (*D–G*) from biological triplicate experiments. ∗∗*p* < 0.01; NS, not significant; Mann-Whitney test for (*D* and *E*); two-way ANOVA for (*F* and *G*). HMECs, human mammary epithelial cells; HTATSF1, HIV Tat-specific factor 1.

To assess the function of the CK2–HTATSF1–TOPBP1 axis in breast tumors, we examined the expression of HTATSF1 and TOPBP1, as well as the level of HTATSF1 pS748 in an immortalized but nontransformed human breast epithelial cell MCF10A, human mammary epithelial cells (HMECs), and breast cancer cells of different molecular subtypes, including T-47D, SK-BR-3, BT-549, and MDA-MB-231 cells. Immunoblotting analysis showed that breast cancer cells expressed higher levels of HTATSF1 and TOPBP1 compared to MCF10A and HMECs, and HTATSF1 pS748 was also upregulated in these cancer cells (Fig. 3*A*). We then confirmed in MDA-MB-231, BT-549, and T-47D cells that both HTATSF1–TOPBP1 binding and S748 phosphorylation were also controlled by CK2 kinase activity by using CK2 inhibitor treatment (Figs. 3*B* and S3*A*), and the HTATSF1–TOPBP1 association is abolished by HTATSF1/S748A mutation, whereas it is maintained by HTATSF1/S748D, which mimics pS748 (Fig. 3*C* and S3*B*), suggesting an integrity of the CK2–HTATSF1–TOPBP1 axis in breast cancer cells.

To further investigate the role of the CK2–HTATSF1–TOPBP1 axis, MDA-MB-231 cells stably expressing HTATSF1/Wt, HTATSF1/S748A, and HTATSF1/S748D were established. In contrast to HTATSF1/Wt and HTATSF1/S748D, HTATSF1/S748A mutation could not efficiently rescue HTATSF1 depletion-induced defects, upon cisplatin (Fig. 3, *D* and *E*) and irradiation (Fig. S3, *C* and *D*) treatment, including TOPBP1 recruitment and RAD51 loading at γH2AX-marked damaged chromatin (Fig. S3*E*) in EdU-labeled S-phase breast cancer cells. Similar observations were obtained using CK2 inhibitor (Fig. S3, *F* and *G*). The impaired TOPBP1 recruitment in EdU-negative cells is perhaps because CK2 inhibition disrupts the assembly of other TOPBP1-scaffolded protein complexes, thereby affecting non-S-phase TOPBP1 loading (12).

Survival analysis revealed that HTATSF1-depleted breast cancer cells were more susceptible to chemotherapeutic treatment, including cisplatin, CPT, rucaparib, and talazoparib (Fig. 3*F*), whereas the additive effect was not detected in HMECs (Fig. 3*G*), indicating that HTATSF1 loss resulted in tumor cell–specific vulnerability to chemotherapeutic drugs. Meanwhile, HTATSF1/S748A could not compensate for the sensitivity of HTATSF1-deficient cells to chemotherapeutic drugs (Fig. 3*F*). These data suggest that the genotoxic prevention effect of HTATSF1 in breast cancer cells relies on the CK2–HTATSF1–TOPBP1 axis.

### CK2–HTATSF1–TOPBP1 axis confers insensitive response of xenograft breast tumor to chemotherapy

**Figure 4:**
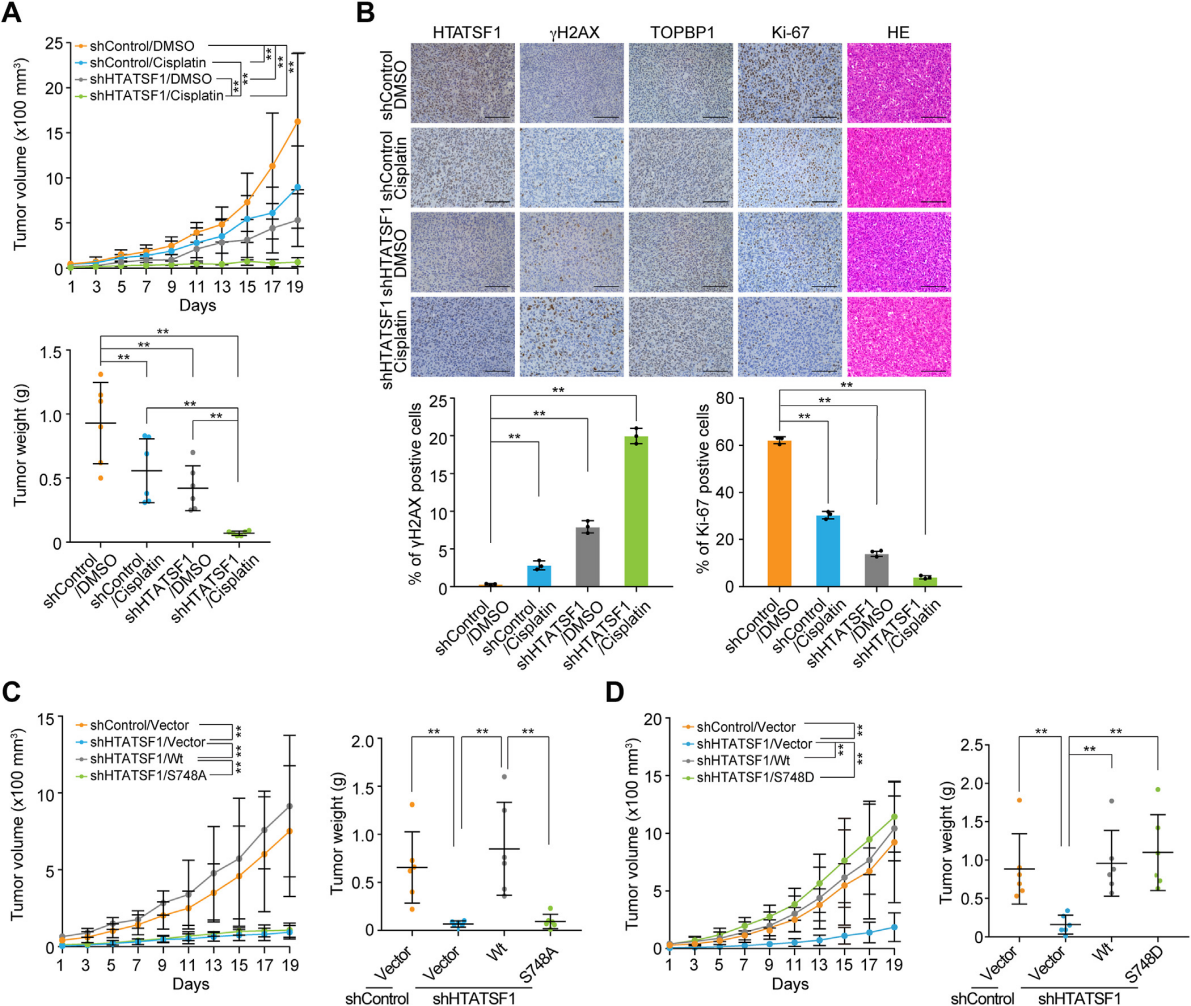
**Hyperactivation of the CK2–HTATSF1–TOPBP1 axis contributes to breast tumorigenesis and confers chemo-insensitivity to breast tumors.***A*, tumor growth and tumor weight of xenografts derived from control or HTATSF1-knockdown MDA-MB-231 cells. Tumor-bearing SCID mice were treated with DMSO or cisplatin every 2 days. *B*, IHC analysis of the levels of HTATSF1, TOPBP1, Ki-67, and γH2AX with samples from (*A*). At least eight fields per tumor (n = 3) of Ki- 67^+^ area and γH2AX^+^ area were analyzed. Representative images are shown. Scale bar, 100 μm. *C*, tumor growth and tumor weight of xenografts from MDA-MB-231 cells stably expressing shHTATSF1, shHTATSF1/Wt, and shHTATSF1/S748A. *D*, tumor growth and tumor weight of xenografts derived from MDA-MB-231 cells stably expressing shHTATSF1, shHTATSF1/Wt, and shHTATSF1/S748D. Tumor-bearing SCID mice were treated with cisplatin every 2 days. Data are mean ± SD for (*A–D*). ∗∗*p* < 0.01; two-way ANOVA for the top panels of (*A*) and *left panels* of (*C*) and (*D*); one-way ANOVA for (*B*), the bottom panels of (*A*) and *right panels* of (*C* and *D*). HTATSF1, HIV Tat-specific factor 1; IHC, immunohistochemistry; SCID, severe combined immunodeficiency.

To further investigate the clinical relevance of the CK2–HTATSF1–TOPBP1 axis in breast tumorigenesis and chemotherapy, stable shControl and shHTATSF1 MDA-MB-231 breast cancer cells were orthotopically transplanted into the mammary fat pads of 6-week-old immunocompromised female severe combined immunodeficiency (SCID) mice followed by DMSO or cisplatin treatment. The results showed that either *HTATSF1* knockdown or cisplatin treatment limited tumor growth, while the combined treatment elicited a synergistic therapeutic effect (Fig. 4*A*). Consistent with the inhibition of tumor growth, we observed accumulation of DNA damage as indicated by γH2AX and reduction of cell proliferation as indicated by Ki-67 in *HTATSF1*-depleted tumors, and the alterations of these indicators were more pronounced under further combined treatment with cisplatin (Fig. 4*B*).

Next, shRNA-resistant HTATSF1/Wt, HTATSF1/S748A, and HTATSF1/S748D were individually stably transfected into HTATSF1-depleted MDA-MB-231 cells, and the tumor formation of these cancer cells was assessed using a cisplatin-challenged xenograft model. The results showed that HTATSF1/Wt and HTATSF1/S748D, but not HTATSF1/S748A, were able to compensate for the synthetic lethal effects associated with HTATSF1 deficiency (Fig. 4, *C* and *D*). Taken together, these results support the argument that a hyperactivated CK2–HTATSF1–TOPBP1 axis confers poor responsiveness of breast tumors to chemotherapeutic agents and that loss of HTATSF1 renders breast tumors more vulnerable to cisplatin treatment.

### Breast tumors with deleted or truncated mutations of HTATSF1 are hypersensitive to chemotherapeutics

**Figure 5:**
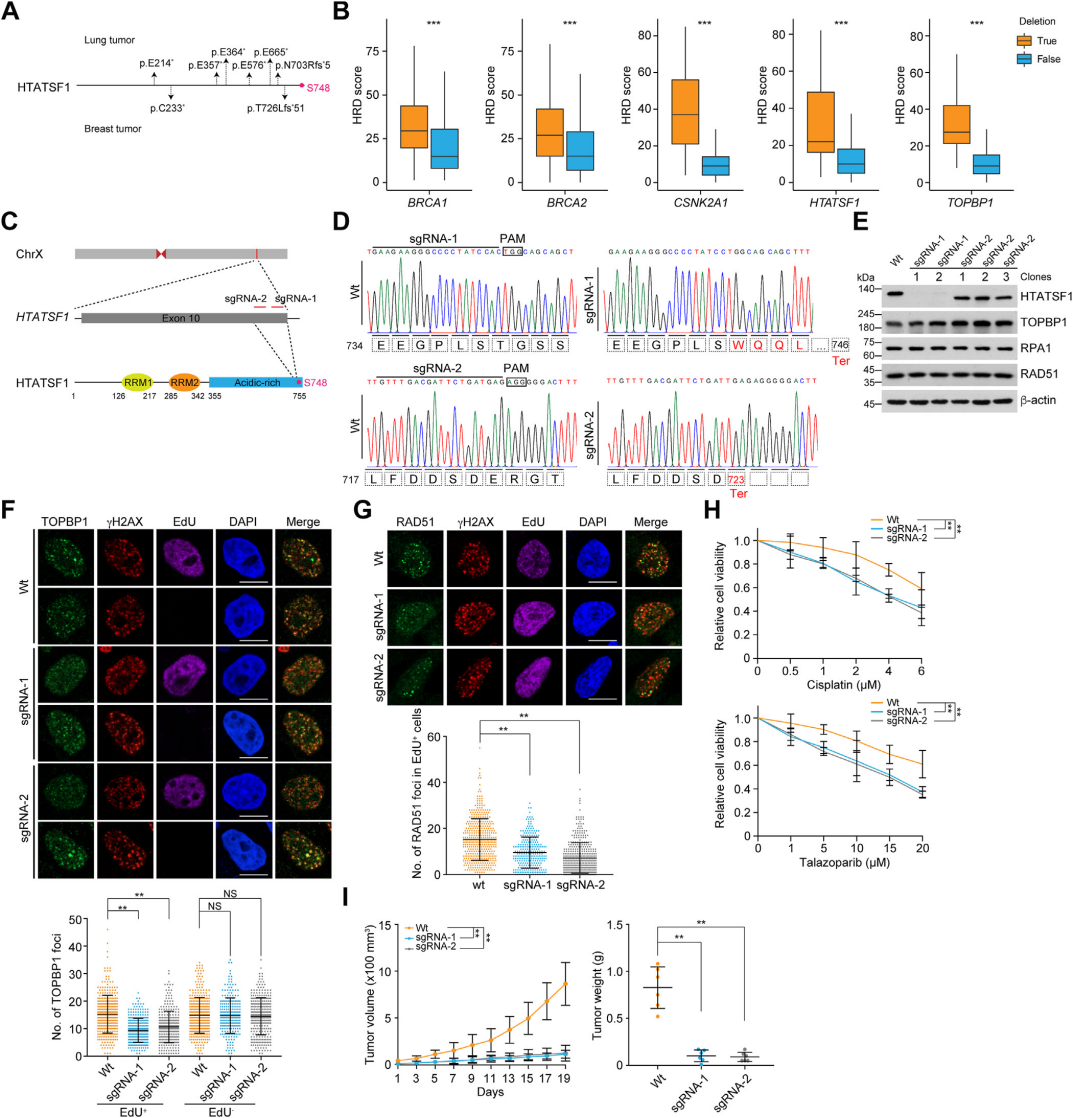
**Deleted or truncated mutations of *HTATSF1* confer breast tumors susceptible to chemotherapeutic drugs.***A*, summary of nonsense substitutions and frameshift deletions occurring in front of S748 codon on *HTATSF1* in breast and lung tumors. *B*, the homologous recombination deficiency (HRD) scores of TCGA breast tumors with genomic deletions of *BRCA1*, *BRCA2*, *CSNK2A1*, *HTATSF1*, or *TOPBP1*. Samples with *BRCA1* deletions are excluded from the *CSNK2A1*, *HTATSF1*, and *TOPBP1* group. *C*, schematic representation of the genome structure of *HTATSF1* and the genomic loci of single-guide (sg) RNAs and their targeting region on HTATSF1 protein. *D*, DNA sequencing results of CRISPR/Cas9-edited MDA-MB-231 cells by two different sgRNAs from single colony. *E*, immunoblotting analysis of HTATSF1 expression in CRISPR/Cas9-edited MDA-MB-231 cells by different sgRNAs. Single colony edited by each sgRNA was analyzed. *F*, immunostaining and confocal microscopy analysis of TOPBP1 foci formation in CRISPR/Cas9-edited MDA-MB-231 cells. Two (sgRNA-1) or three (sgRNA-2) single colonies for each sgRNA-edited cell were pooled and cultured. Cells were irradiated (6 Gy) and labeled with EdU for 1 h, followed by pre-extraction and fixation. The foci number in EdU-positive and EdU-negative cells was quantified (n > 220). Scale bar, 10 μm. *G*, immunostaining and confocal microscopy analysis of RAD51 foci formation in CRISPR/Cas9-edited MDA-MB-231 cells. Cells were irradiated (6 Gy) and labeled with EdU for 1 h before collection. The foci number in EdU-positive cells was quantified (n > 210). Scale bar, 10 μm. *H*, survival analysis of CRISPR/Cas9-edited MDA-MB-231 cells treated with cisplatin or talazoparib. *I*, tumor growth and tumor weight of xenografts derived from CRISPR/Cas9-edited MDA-MB-231 cells. The SCID mice bearing tumors were treated with cisplatin every 2 days. Data are mean ± SD for (*F–H*) from biological triplicate experiments. ∗∗*p* < 0.01, ∗∗∗*p* < 0.001; Mann-Whitney test for (*F* and *G*); two-way ANOVA for (*H*) and the *left panel* of (*I*); one-way ANOVA for the *right panel* of (*I*). HTATSF1, HIV Tat-specific factor 1; NS, not significant; SCID, severe combined immunodeficiency.

**Figure 6:**
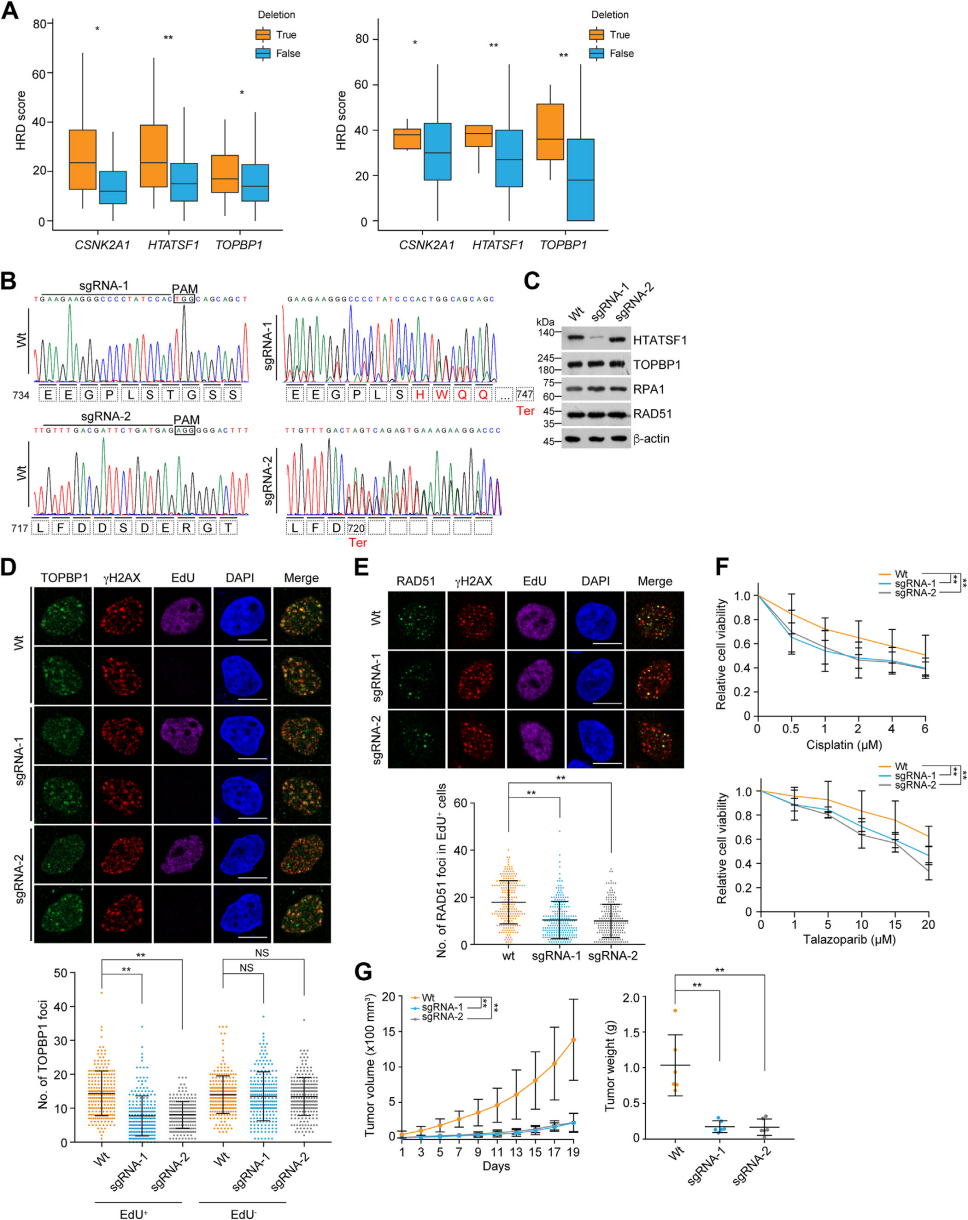
**Deleted or truncated mutations of *HTATSF1* confer lung tumors susceptible to chemotherapy.***A*, the homologous recombination deficiency (HRD) scores of TCGA tumors of LUAD (*left*) and LUSC (*right*) with genomic deletion of *CSNK2A1*, *HTATSF1*, or *TOPBP1*. Samples with *BRCA* deletions in lung tumors are excluded from the *CSNK2A1*, *HTATSF1*, and *TOPBP1* group. *B*, DNA sequencing results of CRISPR/Cas9-edited A549 cells by two different sgRNAs from randomly pooled single colonies. *C*, immunoblotting analysis of HTATSF1 expression in CRISPR/Cas9-edited A549 cells from (*B*) by different sgRNAs. *D*, immunostaining and confocal microscopy analysis of TOPBP1 foci formation in CRISPR/Cas9-edited A549 cells. Cells were irradiated (6 Gy) and labeled with EdU for 1 h, followed by pre-extraction and fixation. The foci number in EdU-positive and EdU-negative cells was quantified (n > 230). Scale bar, 10 μm. *E*, immunostaining and confocal microscopy analysis of RAD51 foci formation in CRISPR/Cas9-edited A549 cells. Cells were irradiated (6 Gy) and labeled with EdU for 1 h before collection. The foci number in EdU-positive cells was quantified (n > 210). Scale bar, 10 μm. *F*, survival analysis of CRISPR/Cas9-edited A549 cells under cisplatin or talazoparib treatment. *G*, tumor growth and tumor weight of xenografts derived from CRISPR/Cas9-edited A549 cells. Tumor-bearing SCID mice were treated with cisplatin every 2 days. Data are mean ± SD for (*D–F*) from biological triplicate experiments. ∗*p* < 0.05, ∗∗*p* < 0.01; Mann-Whitney test for (*D* and *E*); two-way ANOVA for (*F*) and the *left panel* of (*G*); one-way ANOVA for the *right panel* of (*G*). HTATSF1, HIV Tat-specific factor 1; LUAD, lung adenocarcinoma; LUSC, lung squamous cell carcinoma; NS, not significant; SCID, severe combined immunodeficiency.

To further extend the clinical significance of our findings, we analyzed the somatic mutation profile of *HTATSF1* in tumors using the Catalogue of Somatic Mutations In Cancer and TCGA databases. Although no missense mutation corresponds to S748, nonsense substitutions and frameshift deletions in front of the S748 codon occur at a rate of approximately 4% in all types of malignancies, including lung cancer, endometrial cancer, breast cancer, large intestine cancer, and others (Fig. 5*A* and Table S3). Theoretically, all these mutational events will generate premature stop codons and thus truncated proteins or degraded transcripts due to nonsense-mediated decay, both of which will inactivate the CK2–HTATSF1–TOPBP1 axis and allow tumors to respond well to chemotherapeutic drugs. Importantly, we found that a fraction of tumor samples in several types of malignancy carry one or more deleted alleles of *HTATSF1*. For instance, nearly 15% of breast tumor samples from the TCGA datasets have *HTATSF1* deletions, and the number of this event is similar in lung tumors (Fig. 1*D*). Meanwhile, copy number losses of *CSNK2A1* and *TOPBP1*, ranging from 5% to 25%, are also observed in breast, lung, and other tumors (Fig. 1*D*). Similar to *BRCA1* or *BRCA2* deletion, copy number loss of each gene in the CK2–HTATSF1–TOPBP1 axis predicted higher HRD scores in TCGA breast tumors (Fig. 5*B*). Similar results were observed in TCGA lung tumors (Fig. 6*A*). These results suggest that the CK2–HTATSF1–TOPBP1 axis is inactivated in a fraction of clinical tumors, albeit it is generally hyperactivated.

To evaluate the clinical relevance of these mutations and deletions, we first used the CRISPR/Cas9 system to generate a premature stop codon before the coding sequence of HTATSF1 S748 on genomic DNA in breast cancer cells (Fig. 5*C*). Two independent sets of single-guide RNA (sgRNA) were used, and the editing results were confirmed by DNA sequencing using a single colony of MDA-MB-231 cells (Fig. 5*D*). Immunoblotting analysis showed that HTATSF1 expression was lost in sgRNA-1 cells, possibly due to the nonsense-mediated decay effect (25, 26), whereas a truncated form of HTATSF1 was detected in sgRNA-2 cells with an expression level comparable to that of HTATSF1 in wildtype cells (Fig. 5*E*). Two or three individual colonies for each sgRNA-edited cell were then pooled and cultured to examine the effect of *HTATSF1* mutation or deletion. We showed that both sgRNA-1 and sgRNA-2 cells were characterized by a significantly reduced ability to load TOPBP1 and RAD51 (Fig. 5, *F* and *G*) and were more susceptible to cisplatin or talazoparib (Fig. 5*H*), as well as reduced tumor volume or weight (Fig. 5*I*). Notably, xenograft experiments showed that breast tumors expressing the edited *HTATSF1* gene responded more effectively to cisplatin treatment.

### Lung tumors with deleted or truncated mutations of HTATSF1 are hypersensitive to chemotherapeutics

We then took similar strategies to edit the *HTATSF1* gene in human lung carcinoma A549 cells, which carry a higher percentage of truncated mutation and a similar level of *HTATSF1* deletion compared to breast tumor (Table S3 and Fig. 1*D*). The editing efficiency was confirmed by DNA sequencing and immunoblotting with genomic DNA and cellular extracts of A549 cells derived from randomly pooled colonies, respectively (Fig. 6, *B* and *C*). Phenocopying the edited breast cancer cells, we found that TOPBP1 and RAD51 could not be efficiently deposited on damaged chromatin in sgRNA-1 and sgRNA-2 lung cancer cells (Fig. 6, *D* and *E*).

Cell survival and xenograft experiments showed that *HTATSF1* editing rendered lung cancer cells more sensitive to chemotherapeutic agents and reduced tumor volume or weight (Fig. 6, *F* and *G*). Importantly, we noticed that *HTATSF1*-depleted or truncated lung tumor cells exhibited comparable sensitivity to cisplatin, further highlighting the importance of the CK2–HTATSF1–TOPBP1 axis in DNA damage response and repair. Although the experimental models we have generated may not fully reflect the features of clinical tumors, these results suggest that loss-of-function mutations of *HTATSF1*, including nonsense mutations, frameshift mutations, and genomic deletions, are likely to act as *BRCA* mutations and may provide synthetic therapeutic opportunities.

## Discussions

Since a large proportion of tumors have an active HR pathway and tumors with HRD always regain HR, it is important to understand how tumor-associated HR is regulated. We reported that CK2-catalyzed HTATSF1 pS748 is critically involved in breast tumorigenesis, which raises the possibility of combining chemotherapeutic drugs targeting HTATSF1 pS748 or the HTATSF1–TOPBP1 binding surface for the treatment of HTATSF1–TOPBP1 proficient breast tumors. Specifically, the CK2–HTATSF1–TOPBP1 axis is generally hyperactivated in several tumors, possibly due to gene amplification, and gain-of-function of this pathway sustains tumor cell survival in part by counteracting DNA damage. As we and the others have shown that RAD51 loading at damage sites can be mediated by BRCA2-independent mechanisms (11, 27), it will be interesting to investigate whether hyperactivation of the CK2–HTATSF1–TOPBP1 axis could contribute to HR reacquisition in *BRCA2*-deficient breast tumors and whether this type of tumor is synthetically lethal with strategies targeting the CK2–HTATSF1–TOPBP1 axis. Additionally, we also provide proof-of-concept evidence that deletion or truncation mutations of HTATSF1, which occur less frequently in clinical tumors, confer sensitivity to PARPis or platinum drugs.

Our previous study showed that CK2 kinase-mediated pS748 of HTATSF1 facilitates HTATSF1–TOPBP1 association to promote HR repair (12). Therefore, it is necessary to understand the role of the CK2–HTATSF1–TOPBP1 axis in tumor HR repair and the therapeutic implications. Indeed, HTATSF1/S748A, which does not interact with TOPBP1, could not rescue HTATSF1 depletion–induced repair defects and chemotherapeutic sensitivity in breast cancer cells. The synthetic lethality of HTATSF1 loss or S748A mutation with cisplatin was further confirmed by xenograft experiments. Our study argues that targeting this axis in combination with PARPis or platinum drugs will benefit tumor patients with hyperactive CK2–HTATSF1–TOPBP1, and understanding the activity of this pathway may precisely guide the application of the CK2 inhibitor CX-4945, currently in clinical trials for various cancers (23). The hyperactivated CK2–HTATSF1–TOPBP1 axis confers poor responsiveness of breast tumors to chemotherapeutics and may contribute to breast tumorigenesis by coping with endogenous replication stress, although the transcriptional processing activity of HTATSF1 in tumorigenesis cannot be overlooked. We propose that targeting CK2 or disrupting the molecular interface of HTATSF1–TOPBP1 may be a promising therapeutic strategy for the treatment of CK2–HTATSF1–TOPBP1–proficient breast tumors. However, it is necessary to investigate the potential function of HTATSF1–TOPBP1 in other malignancies and whether other effectors may contribute to HTATSF1-regulated breast tumorigenesis.

As we can see, the mRNA levels of *CSNK2A1*, *HTATSF1*, and *TOPBP1* are positively correlated with each other in multiple tumors, suggesting that *CSNK2A1*, *HTATSF1*, and *TOPBP1* may share a convergent mechanism of regulation at the genomic or transcriptional level. Copy number variation analysis showed that *CSNK2A1*, *HTATSF1*, and *TOPBP1* gain copy number and their amplification is tightly associated with expression, suggesting a possible genomic cooption for the three genes during tumor evolution. Consistent with the genomic status of these genes, analysis of clinical tumor samples showed that the level of pS748, which may largely reflect the integrity of the CK2–HTATSF1–TOPBP1 pathway, is positively correlated with the histological grade of breast cancer and predicts survival in patients. Although HTATSF1 pS748 could be a marker of higher proliferation or an indicator of active replication, the functional characterization of this phosphorylation site in our previous report and this study suggests that it plays an important role in promoting tumor progression by favoring TOPBP1-mediated HR repair of damaged replicating chromatin. This suggests that pS748 may serve as a functional biomarker in oncology, and targeting the CK2–HTATSF1–TOPBP1 pathway may guide future therapeutic strategies. However, the extent to which our findings can be applied to breast tumors with highly complex biological heterogeneity needs to be further investigated.

To date, there have been few studies on HTATSF1 in tumorigenesis, and specific inhibitors targeting HTATSF1 are still not available. Here, we identified S748 in the C-terminal acid-rich domain of HTATSF1 as a necessary site for HTATSF1–TOPBP1 interaction and HR repair in breast tumors. Although no missense mutation corresponding to S748 was detected, analysis of the Catalogue of Somatic Mutations In Cancer and TCGA datasets showed that nonsense substitutions and frameshift deletions before the S748 codon occur in several malignancies (4%), which may generate truncated proteins or degraded transcripts. These mutations will inactivate the CK2–HTATSF1–TOPBP1 axis and induce hypersensitivity of breast tumors to chemotherapeutic agents. Although truncated mutations account for only a small proportion of clinical tumor samples, the relatively higher frequency of *HTATSF1* deletion mutations (15%) or copy number loss of *CSNK2A1* and *TOPBP1* (5–25%) may further expand its application as a potential therapeutic strategy for breast and lung tumors. These findings potentially offer PARPi or platinum treatment options for patients suffering from breast and lung tumors harboring truncated or deleted mutations of the CK2–HTATSF1–TOPBP1 axis.

## Experimental procedures

### Antibodies and reagents

The sources of antibodies against the following proteins or post-translational modifications were as follows: γH2AX (9718S, for immunofluorescence [IF] and IHC) from Cell Signaling Technology; FLAG (F3165, for WB and IP) from Sigma; RAD51 (ab133534, for IF) from abcam;γH2AX (05–636, for IF and IHC) from Millipore; HTATSF1 (A302–023A, for WB and IHC) and TOPBP1 (A300–111A, for WB and IP) from Bethyl Lab; β-actin (AC004, for WB) from Abclonal; HTATSF1 pS748 produced by PTM BIO; TOPBP1 (sc-271043, for IF and IHC) from Santa Cruz; and Ki67 (ZA-0502, for human tissue IHC) from ZSGB-BIO. Protein G Magnetic Beads (10004D) was purchased from Invitrogen. Anti-FLAG M2 affinity gel (A2220), CPT (C9911), blasticidin (15205),and puromycin (P8833) were purchased from Sigma. Rucaparib (S1098), talazoparib (S7048), and cisplatin (S1166) were purchased from Selleck.

### Cell culture

MCF-7, HEK 293T, MDA-MB-231, T-47D, BT-549, SK-BR-3, MCF-10A, and HMEC cells were purchased from the American Type Culture Collection and cultured according to the manufacturer’s instructions. All cells were authenticated by examination of morphology and growth characteristics and confirmed to be free of *mycoplasma*.

### Immunofluorescence

Cells on glass coverslips (BD Biosciences) were fixed with 4% paraformaldehyde and permeabilized with 0.2% Triton X-100 in phosphate-buffered saline (PBS). Samples were blocked in 5% donkey serum in the presence of 0.1% Triton X-100 and stained with the appropriate primary and secondary antibodies conjugated to Alexa Fluor 488, 594, or 647 (Invitrogen). Confocal images were captured using a Zeiss LSM 800 microscope with a ×63 oil objective. To avoid bleed-through effects in double-staining experiments, each dye was scanned independently in a multitracking mode. When examining nuclear widespread TOPBP1 foci, cells were pretreated with 0.5% Triton X-100 for 5 min on ice to extract nonchromatin fractions and fixed with 3% paraformaldehyde and 2% sucrose for 15 min at room temperature. Cells were then permeabilized with 0.5% Triton X-100 for 5 min on ice and incubated in blocking buffer (0.1% Triton X-100 and 5% donkey serum in PBS) for 1 h at room temperature. For S-phase discrimination, cells were pulsed with 10 μM EdU at 37 ºC for 1 h prior to fixation. Incorporated EdU was click-labeled with keyFluor 647-azide (Keygen Technologies) according to the manufacturer’s instructions.

### Irradiation

Irradiation was delivered by an X-ray generator (RS2000 PRO, Radsource Corporation). Cells were generally exposed to irradiation (160 kV, 25 mA; 1 Gy/min) for 6 min. After irradiation, the cells were incubated at 37 °C before processing for immunostaining as described previously.

### Tumor xenografts

MDA-MB-231 cells stably expressing control shRNA or *HTATSF1* 3′UTR shRNA were resuspended with 200 μl PBS (1.5 × 10^6^) and orthotopically transplanted onto the mammary fat pad of 6-week-old immunocompromised female SCID mice. When tumor volume reached 40 mm^3^, mice were randomly divided in half and treated with cisplatin (2.5 mg/kg, 5% v/v DMF, 40% v/v PEG300, and 2% v/v Tween80 in H_2_O) or DMSO every 2 days. Six animals per group were used for each experiment. Tumors were measured every 2 days by using a vernier caliper, and the volume was calculated using the formula: 0.5 × length × width^2^. For rescue experiments, MDA-MB-231 cells were subsequently infected with lentivirus expressing FLAG-tagged HTATSF1 variants and *HTATSF1* 3′UTR shRNA followed by puromycin selection and fluorescence-activated cell sorting. Similar strategies were used for xenograft experiments with MDA-MB-231 or A549 cells expressing truncated or deleted *HTATSF1*. All studies were approved by the Animal Care Committee of Tianjin Medical University.

### HTATSF1 truncation or deletion cell generation

HTATSF1 truncation or deletion cells were generated by cotransfection of plasmid encoding FLAG-Cas9 (lentiCas9-Blast) and sgRNA plasmid (lentiGuide-Puro) targeting HTATSF1 (sgRNA-1: GAAGAAGGGCCCCTATCCAC, sgRNA-2: TGTTTGACGATTCTGATGAG). Forty-eight hours after transfection, the cells were selected with blasticidin (5 ug/ml) and puromycin (1 μg/ml) for 2 days. Single colonies were sorted and analyzed by flow cytometry.

### Immunoprecipitation

Cell extracts were prepared using NETN buffer (50 mM Tris-HCl, pH 8.0, 150 mM NaCl, 0.2% NP-40, and 2 mM EDTA) supplemented with protease inhibitors (Roche) for 20 min, then centrifuged at 14,000*g* for 15 min at 4 °C. For immunoprecipitation, ∼500 μg protein was incubated for 12 h at 4 °C with 1 to 2 μg control or specific antibodies, then 50 μl of 50% protein G magnetic beads (Invitrogen) were added and incubated for a further 2 h. The beads were washed five times using lysis buffer and collected on a magnetic stand (Invitrogen) at 4 °C. Precipitated proteins were eluted from the beads using 2 × SDS-PAGE loading buffer and then analyzed with Western blotting.

### Cell survival assay

Cells were plated into 96-well plates at a density of 2000 cells per well for 24 h and then treated with different doses of genotoxic agents for 72 h. Cell Titer aqueous one solution (G3582, Promega) was added to each well according to the manufacturer’s instructions. After 1 h of incubation, cell viability was determined by measuring the absorbance at 490 nm using a Bio-Rad plate reader (model 550; Bio-Rad).

### RNA interference

HTATSF1 siRNA was transfected using Lipofectamine RNAi MAX (Invitrogen) according to the manufacturer’s instructions. After 72 h of transfection, cells were harvested for experiments. Control siRNA (ON-TARGETplus Non- Targeting Pool, D-001810-10) was got from Dharmacon in a smart pool manner. HTATSF1 siRNA (siRNA-1: CAUAGUGGUAGGAUGCCAU and siRNA-2: GAGUAUUGAUUGAAGUUUG) were chemically synthesized by Sigma.

### Lentiviral production

For *HTATSF1* gene silencing, *HTATSF1* 3′UTR shRNA (shRNA: GAGTATTGATTGAAGTTTG) was cloned into pLB lentiviral vector (Addgene, 11619). The lenti vector then was cotransfected with pMDLg/pRRE, pRSV-REV, and pVSVG into HEK 293T cells. After 48 h of transfection, viral supernatants were collected and clarified by filtration. We then infected MDA-MB-231 cells with these lentivirus and sorted the EGFP-positive cells by fluorescence-activated cell sorting analysis.

### Immunohistochemistry

IHC was performed on xenograft tissues and paraffin-embedded tissues (Shanghai Xinchao Biological Technology). Briefly, tissue sections were deparaffinized with xylene and rehydrated through a gradient ethanol immersion. Endogenous peroxidase activity was quenched by 3% (vol/vol) hydrogen peroxide in methanol for 30 min, followed by three 3-min washes with PBS. The slides were then immersed in 0.01 M citrate buffer solution (pH 6.0) and placed in a microwave oven for 30 min. After washing in PBS, the sections were blocked with 10% (v/v) normal goat serum for 30 min and incubated overnight in a humidity chamber at 4 °C with the primary antibody diluted in 10% normal goat serum. After three 5-min washes with PBS, sections were treated with a peroxidase-conjugated secondary antibody (ZSGB-BIO) for 30 min at room temperature, followed by three additional 5-min washes with PBS. DAB solution (ZSGB-BIO) was added, and the slides were counterstained with hematoxylin according to the manufacturer’s instructions. Images were captured using an Olympus VS120 Slide Scanner, and immunohistochemical staining was evaluated by independent pathologists. The total protein expression score was calculated by multiplying the percentage of immune-positive areas by the immunostaining intensity. Prognostic significance for overall survival was estimated based on the Cox proportional hazards regression model using the ‘survival’ R package (version 3.5).

### Antibody production

Antibodies against HTATSF1/pS748 were designed and produced by PTM Biolabs. Briefly, an antigenic peptide (CSFILS-(phosphor)S-DDDDD) was synthesized and conjugated with keyhole limpet hemocyanin for immunization of rabbits. To prepare the immune material, the immunogen was diluted with normal saline and mixed 1:1 with the appropriate adjuvant to form a stable emulsion. The antigen mixture was extracted with a syringe and injected subcutaneously into two sites on the shoulders and the hind leg muscles of rabbits (approximately one-fourth volume of immunogen per site). This allowed the immunogen to persist and thus enhance the immune response. There were four immunizations for each rabbit, on day 1, day 21, day 28, and day 35. Blood was then collected as follows: first blood was collected on the 45th day, and 30 ml of whole blood was collected for ELISA and dot blot analysis with the supernatant and second/third/fourth blood was collected on the 50th, 65th, and 70th days, 20 ml each time, and the supernatant was collected for serum screening including ELISA and dot blot. The positive antiserum was used for further purification. After the serum was filtered, the sample was applied to a well-balanced protein A chromatography column. The effluent of the sample was retained to determine the binding efficiency of the antiserum and the packing. The columns were washed with a PBS buffer solution and eluted with 150 mM glycine buffer solution. The eluent was collected and added to the neutralization buffer to adjust the pH to 7.0. The coarse pure IgG purified by the Protein A column was balanced against the affinity chromatography column conjugated to specific antigenic peptides to enrich the antibody. The effluent was then eluted against the affinity chromatography column conjugated to unmodified peptides to remove the unspecific antiserum, and the final eluent was collected and stored in 10% glycerol at 4 °C.

### RNA-seq analysis

The copy number profiles and RNA-seq data (measured as fragments per kilobase of transcript per million mapped reads) for the TCGA cohorts were obtained from the Genomic Data Commons (28) at https://portal.gdc.cancer.gov. The information on gene expression and amplification from these datasets has been provided in Tables S1 and S2. The HRD score data were obtained from previously published data (29). Human breast tumor RNA-seq data were downloaded from GEO using accession no. GSE109169, GSE45827, and GSE65194. The ggplot2 (version 3.4.2) and pheatmap (version 1.0.12) packages were used to analyze the data. The correlation between gene expression levels was determined as the Pearson correlation coefficient. All statistical analyses and visualizations were performed using R (version 4.2.0). Unpaired two-tailed Student’s *t* test was used for a significant test of expression difference between the indicated two groups. ∗*p* < 0.05; ∗∗*p* < 0.01; ∗∗∗*p* < 0.001.

### Quantification and statistical analysis

Immunofluorescence foci were counted by ImageJ. Colocalization analyses were done in ImageJ. All statistical analyses were performed by Prism GraphPad. Data from biological triplicate or duplicate experiments are presented as mean ± SD. Two-tailed unpaired Student *t* test was used to compare two groups of data. ANOVA with Bonferroni’s correction was used to compare multiple groups of data. In box plots, values that are less than or equal to the first quartile minus 1.5 times the interquartile range or greater than or equal to the third quartile plus 1.5 times the interquartile range are defined as outliers and marked with a circle. The Mann-Whitney U test or chi-squared test was used for non-normally distributed values. *p* < 0.05 was considered statistically significant. Before statistical analysis, the variation within each group of data and the assumptions of the tests were checked.

## Data availability

All data needed to evaluate the conclusions are presented in the paper.

## Supporting information

This article contains supporting information.

## Conflict of interest

The authors declare that they have no conflicts of interest with the contents of this article.

## Figures and Tables

**Supplemental Table S1:**