# Supplementary material for: CK2–HTATSF1–TOPBP1 signaling axis modulates tumor chemotherapy response
Source: J Biol Chem. 2024 May 16;300(6):107377. doi: 10.1016/j.jbc.2024.107377 (PMC11208909; doi:10.1016/j.jbc.2024.107377)
Supplement: Supplemental Figures S1–S3 [file mmc1.docx]

**CK2-****HTATSF1-TOPBP1 signaling axis modulates tumor chemotherapy response**

Qiushi Guo^1, 3^, Jiao Zhao^2, 3^, Yuan Li^1^, Chunyong Zhang^1^, Xilin Shen^1^, Ling Liu^1^, Zhenzhen Yang^1^, Shuai Ma^1, #^, Yan Qin^1, #^, Lei Shi^1, #^

^1^Key Laboratory of Breast Cancer Prevention and Therapy (Ministry of Education), Key Laboratory of Immune Microenvironment and Disease (Ministry of Education), The Province and Ministry Co-sponsored Collaborative Innovation Center for Medical Epigenetics, School of Basic Medical Sciences, Tianjin Medical University Cancer Institute and Hospital, Tianjin Medical University, Tianjin 300070, China;

^2^Department of Clinical Laboratory, Key Clinical Laboratory of Henan Province, The First Affiliated Hospital of Zhengzhou University, Zhengzhou 450052, China;

^3^These authors contributed equally to this work.

^#^Correspondence: Lei Shi, Ph.D.; Yan Qin, Ph. D., Shuai Ma, Ph. D..

E-mail: [shilei@tmu.edu.cn](mailto:shilei@tmu.edu.cn)

[qinyan1@tmu.edu.cn](mailto:qinyan1@tmu.edu.cn)

mashuai@tjmuch.com

**Supporting Information**
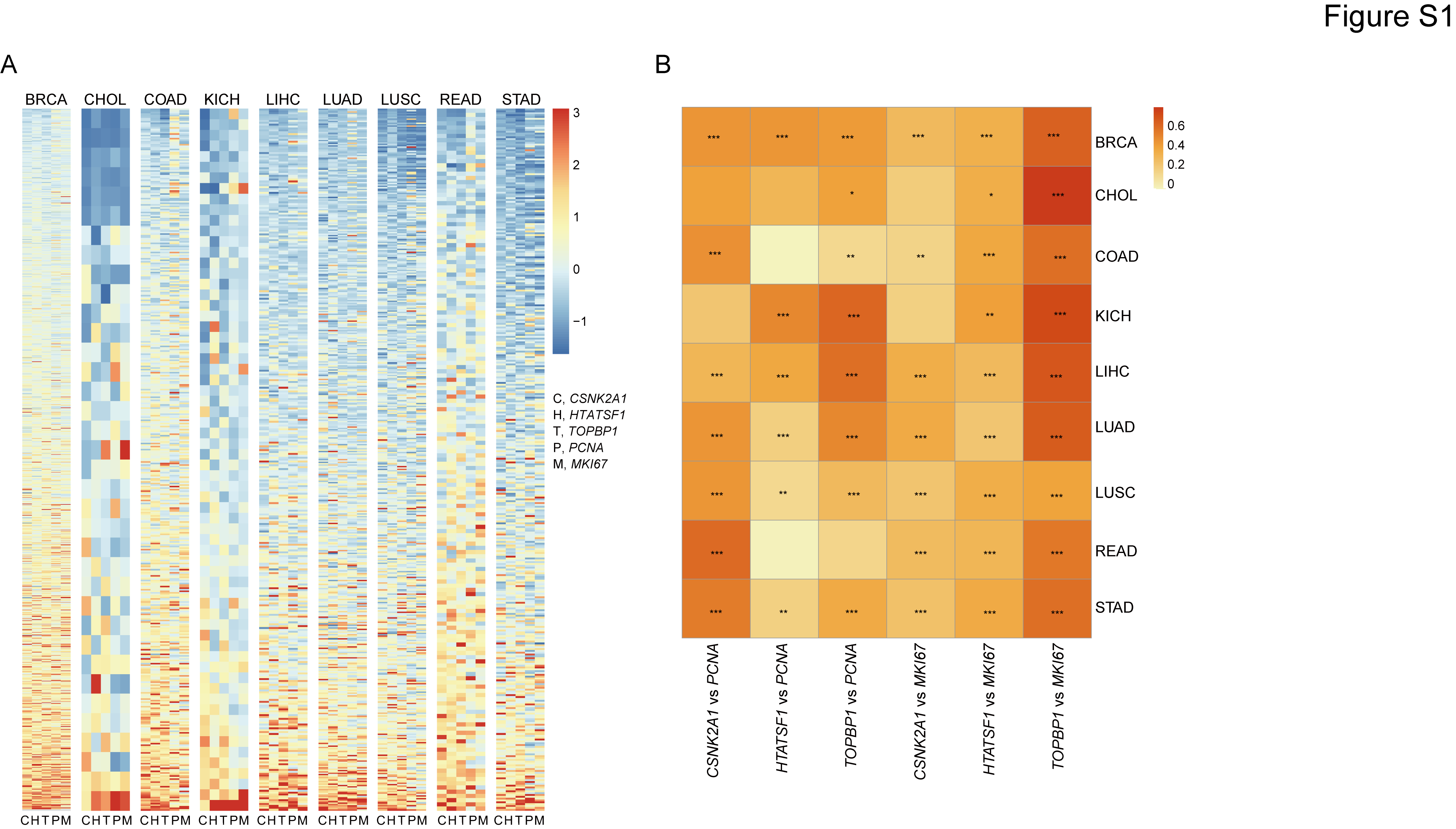


**Figure S1 (Figure 1 continued). The correlation of *CSNK2A1*, *HTATSF1*, and *TOPBP1* with proliferation signatures in breast tumor samples.** (A) The mRNA expression profile of *CSNK2A1*, *HTATSF1*, *TOPBP1*, *PCNA*, and *MKI67* in 9 tumor types as indicated by TCGA datasets. The color key of the Z-score represents the normalized expression. (B) The mRNA expression correlation between each two of *CSNK2A1*, *HTATSF1*, *TOPBP1*, *PCNA*, and *MKI67* in 9 types of TCGA tumors. The color key represents Pearson correlation coefficient. **P* < 0.05, ***P* < 0.01, and ****P* < 0.001 were analyzed by two-tailed unpaired Student’s t-test.

**
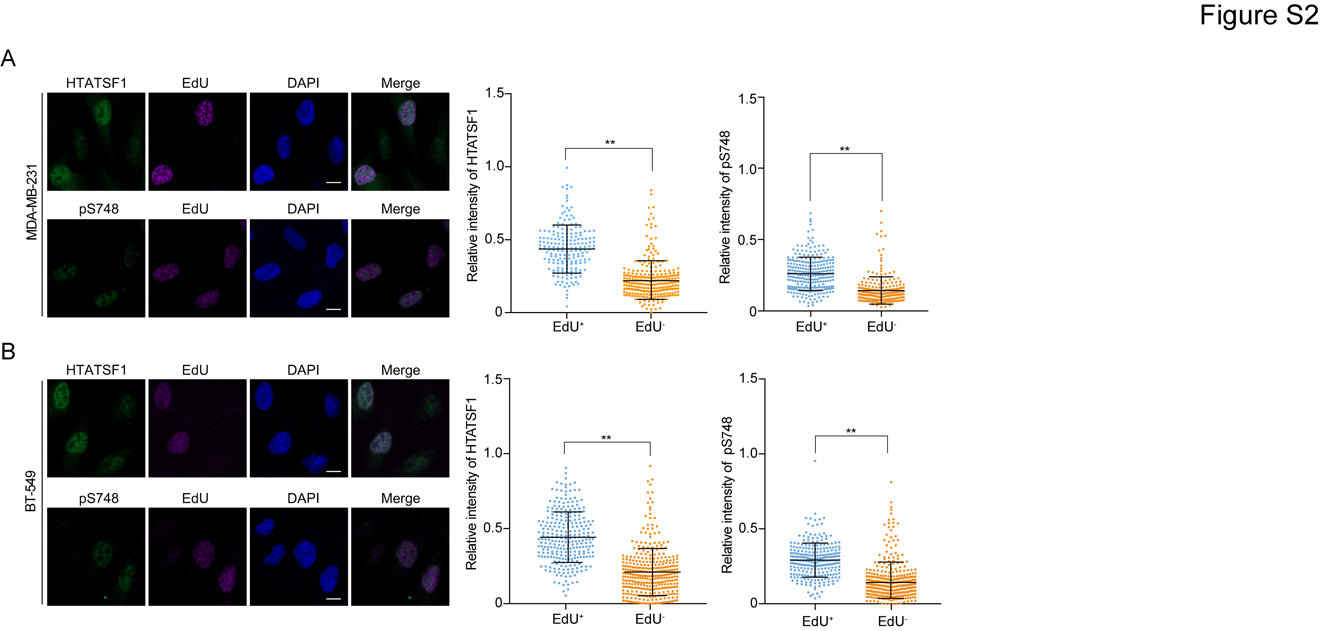
**

**Figure S2 (Figure 2 continued). The expression of HTATSF1 and pS748 is upregulated in EdU-positive breast tumor cells.** (A) Immunofluorescence and confocal microscopy analysis of HTATSF1 and HTATSF1 pS748 in EdU-labelled MDA-MB-231 cells. The intensity of HTATSF1 and pS748 in EdU-positive and EdU-negative cells was quantified and normalized against that of DAPI signal (n > 200). Scale bar, 10 μm. (B) Immunofluorescence and confocal microscopy analysis of HTATSF1 and HTATSF1 pS748 in EdU-labelled BT-549 cells. The intensity of HTATSF1 and pS748 in EdU-positive and EdU-negative cells was quantified and normalized against that of DAPI signal (n > 200). Scale bar, 10 μm.

Data are mean ± SD for (A)-(B). ***P* < 0.01.Mann-Whitney test for (A)-(B).

**
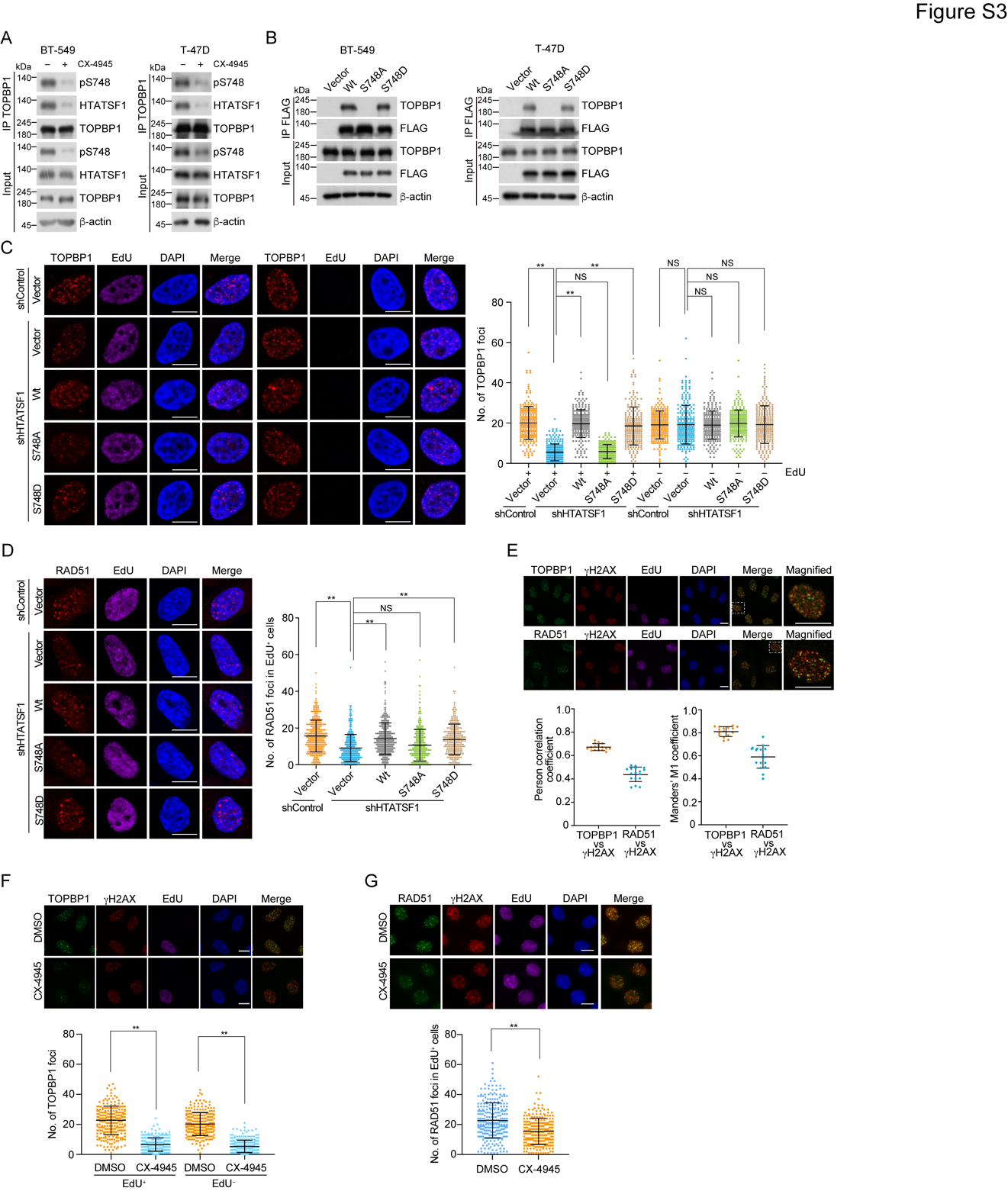
**

**Figure S3 (Figure 3 continued). CK2-HTATSF1-TOPBP1 axis protects breast cancer cells from genotoxic insults.** (A) Immunoprecipitation followed by immunoblotting with cellular extracts from BT-549 and T-47D cells treated with CK2 inhibitor CX-4945 (10 μM, 4 hours). (B) Co-immunoprecipitation analysis of the interaction between endogenous TOPBP1 and the indicated point mutants of FLAG-tagged HTATSF1 in BT-549 and T-47D cells. (C) Immunostaining and confocal microscopy analysis of TOPBP1 foci formation in MDA-MB-231 cells. Cells stably expressing HTATSF1 3’UTR shRNA and HTATSF1/Wt, HTATSF1/S748A, or HTATSF1/S748D, were irradiated (6 Gy) and labelled with EdU for 1 hour followed by pre-extraction and fixation. The foci number in EdU-positive and EdU-negative cells was quantified (n > 200). (D) Immunostaining and confocal microscopy analysis of RAD51 foci formation in MDA-MB-231 cells. Cells stably expressing HTATSF1 3’UTR shRNA and HTATSF1/Wt, HTATSF1/S748A, or HTATSF1/S748D, were irradiated (6 Gy) and labelled with EdU for 1 hour. The foci number in EdU-positive cells was quantified (n > 200). (E) Immunostaining analysis of the colocalization of γH2AX and TOPBP1 or RAD51 in EdU-labelled MDA-MB-231 cells. Cells were irradiated (6 Gy) followed by pre-extraction and fixation. The colocalization signal was quantified using Pearson correlation coefficient and Manders’ M1 coefficient analysis. More than 200 cells from 16-17 fields of the images were quantified. Pearson correlation and Manders’ M1 coefficient (-1~1), 1 represents perfect correlation, -1 represents complete exclusion, and 0 represents a random relationship. (F) Immunostaining and confocal microscopy analysis of TOPBP1 foci formation in MDA-MB-231 cells. Cells were treated with 10 μM CX-4945 for 4 hours and labelled with EdU for 1 hour followed by pre-extraction and fixation. The foci number in EdU-positive and EdU-negative cells was quantified (n > 200). (G) Immunostaining and confocal microscopy analysis of RAD51 foci formation in MDA-MB-231 cells. Cells were treated with 10 μM CX-4945 for 4 hours and labelled with EdU for 1 hour. The foci number in EdU-positive cells was quantified (n > 200). Scale bar, 10 μm.

Data are mean ± SD for (C)-(G) from biological triplicate experiments. NS, not significant; ***P* < 0.01. Mann-Whitney test for (C)-(D) and (F)-(G).
